# Randomised controlled trial of analgesia for the management of acute severe pain from traumatic injury: study protocol for the paramedic analgesia comparing ketamine and morphine in trauma (PACKMaN)

**DOI:** 10.1186/s13049-023-01146-1

**Published:** 2023-11-24

**Authors:** F. Michelet, M. Smyth, R. Lall, H. Noordali, K. Starr, L. Berridge, J. Yeung, G. Fuller, S. Petrou, A. Walker, J. Mark, A. Canaway, K. Khan, G. D. Perkins

**Affiliations:** 1 Warwick Clinical Trials Unit, University of Warwick, Coventry, UK; 2 School of Health and Related Research, University of Sheffield, Sheffield, UK; 3 Nuffield Department of Primary Care Health Sciences, University of Oxford, Oxford, UK; 4 West Midlands Ambulance Services NHS Trust, Brierley Hill, Dudley, UK; 5 Yorkshire Ambulance Services NHS Trust, Wakefield, UK; 6 Critical Care Directorate, University Hospitals Birmingham NHS Foundation Trust, Birmingham, UK

**Keywords:** Traumatic injury, Prehospital analgesia, Ketamine, Morphine

## Abstract

**Background:**

Prehospital analgesia is often required after traumatic injury, currently morphine is the strongest parenteral analgesia routinely available for use by paramedics in the United Kingdom (UK) when treating patients with severe pain. This protocol describes a multi-centre, randomised, double blinded trial comparing the clinical and cost-effectiveness of ketamine and morphine for severe pain following acute traumatic injury.

**Methods:**

A two arm pragmatic, phase III trial working with two large NHS ambulance services, with an internal pilot. Participants will be randomised in equal numbers to either (1) morphine or (2) ketamine by IV/IO injection. We aim to recruit 446 participants over the age of 16 years old, with a self-reported pain score of 7 or above out of 10. Randomised participants will receive a maximum of 20 mg of morphine, or a maximum of 30 mg of ketamine, to manage their pain. The primary outcome will be the sum of pain intensity difference. Secondary outcomes measure the effectiveness of pain relief and overall patient experience from randomisation to arrival at hospital as well as monitoring the adverse events, resource use and cost-effectiveness outcomes.

**Discussion:**

The PACKMAN study is the first UK clinical trial addressing the clinical and cost-effectiveness of ketamine and morphine in treating acute severe pain from traumatic injury treated by NHS paramedics. The findings will inform future clinical practice and provide insights into the effectiveness of ketamine as a prehospital analgesia.

*Trial registration*: ISRCTN, ISRCTN14124474. Registered 22 October 2020, https://www.isrctn.com/ISRCTN14124474

## Background

At least 70% of Ambulance calls involve patients experiencing pain [1]. NHS Paramedics have a limited formulary to treat severe pain. Observational studies suggest that current treatments leave many patients with inadequate pain relief in the prehospital environment [2–6].

Effective management of acute pain is important for humanitarian reasons, for improving patient experience and reducing adverse long-term outcomes. In 2004 the World Health Organisation declared that effective management of pain is a universal human right. Poorly managed acute pain is associated with increased chronic pain. Studies indicate chronic pain is common following trauma with a reported incidence of 15–30%, increasing to 62% in patients suffering major trauma [7–9]. Poorly managed postoperative pain leads to persistent pain in 10–50% of common surgeries, and that pain is severe in about 2–10% of these patients [10]. Military personnel injured in recent conflicts demonstrate a link between acute pain management and subsequent depression or post-traumatic stress disorder (PTSD). Early aggressive pain management exerts a protective effect on the development of PTSD (OR 0.47 (95%CI 0.34–0.66) and depression (0.40 (95% CI 0.17–0.94) [11, 12]. Provision of early and effective analgesia has the potential to reduce the risk of developing chronic pain and adverse mental health outcomes post trauma, which may impact on patient’s long term quality of life [13, 14].

The Joint Royal Colleges Ambulance Liaison Committee (JRCALC) produce national clinical guidelines for NHS Ambulance Services. These guidelines suggest a stepwise approach to pain management according to the pain severity and availability of pre-hospital treatments for pain. The strongest parenteral analgesia routinely available for use by paramedics when treating patients with severe pain is morphine. Ketamine may be an ideal prehospital analgesic agent due to its rapid onset of action, superior analgesic properties and haemodynamic profile. The National Institute for Health and Care Excellence (NICE) has identified the need for a pragmatic, randomised trial to determine the clinical and cost effectiveness of ketamine against standard care (morphine) [15].

## Trial rationale

A barrier to effective pain treatment is the limited formulary available to paramedics. The most frequently used drug for moderate to severe pain outside a hospital is morphine [16]. Unfortunately morphine has several side effects (nausea, confusion, dizziness, drowsiness, respiratory depression, arrhythmia) that may limit its use [17–20]. This, and concerns about potential longer term dependence, limits effective use by clinicians [21]. Ketamine is perceived by many to be an ideal prehospital analgesic agent, favoured for its rapid onset of action, effective analgesia, good haemodynamic stability, and preservation of upper airway reflexes [22]. Ketamine has a distinct dose–response gradient in which small doses (< 0.5 mg/kg) provide an analgesic effect and large doses (> 2 mg/kg) an anaesthetic effect [23]. It exerts its effect by “disconnecting” the thalamocortical and limbic systems, effectively dissociating the central nervous system (CNS) from outside stimuli (e.g. pain, sight, sound) [24]. Ketamine also stimulates the sympathetic nervous system and moderately increases heart rate and blood pressure. Ketamine seldomly affects respiration; patients breathe spontaneously and maintain airway control [25]. Furthermore, there is evidence to indicate that perioperative ketamine analgesia may prevent hyperalgesia, reducing the risk of developing persistent post-operative pain [26, 27]. This suggests the potential for ketamine analgesia to be associated with a lower incidence of chronic pain post trauma.

Ketamine has a wide margin of safety. Serious adverse outcomes have not been reported even though overdoses of 5 to 100 times the intended dose have been inadvertently administered [28]. Due to its rapid onset and favourable side effect profile, ketamine is used in ambulance systems around the world [29–34] There are few definitive trials which compare its effectiveness, safety and cost-effectiveness.

## Methods and analysis

### Aim

The primary aim of this trial is to determine whether paramedic administered ketamine (intervention) or morphine (comparator) provides more effective pain relief for patients reporting severe pain following trauma, as measured by the Sum of Pain Intensity Difference (SPID), assessed using a 0–10 numeric rating scale.

Core trial information is presented in Table 1.

**Table 1:** WHO trial registration data set

Data Category	Information
Primary registry and trial identifying number	ISRCTN14124474
Date of registration in primary registry	22/10/2020
Secondary identifying numbers	EudraCT number: 2020-000154-10 IRAS ID: 1003404 CPMS ID: 46938 REC reference: 20/WS/0126
Source of monetary or material support	National Institute for Health Research (NIHR)
Primary sponsor	University of Warwick
Secondary sponsor	N/A
Contact for public queries	packman@warwick.ac.uk
Contact for scientific queries	m.a.smyth@warwick.ac.uk
Public title	PACKMaN
Scientific title	Paramedic Analgesia Comparing Ketamine and MorphiNe in trauma: PACKMaN
Countries of recruitment	UK
Health condition or problem studied	Acute severe pain from traumatic injury in adults
Interventions	Control: Pre-hospital morphine sulphate (0.10 mg/kg) Intervention: Pre-hopsital ketamine hydrochloride (0.15 mg/kg)
Key inclusion and exclusion criteria	*Inclusion*: Age ≥ 16 Patient reports a pain score ≥ 7/10 on a 0–10 numeric rating scale following acute traumatic injury Intravenous (IV) or intraosseous (IO) access obtained Determined by a paramedic to require IV morphine or equivalent *Exclusion*: Known or suspected pregnancy
Unable to articulate severity of pain using the 0–10 numeric rating scale	
Lack of capacity due to a reason other than pain IV/IO ketamine or opioid analgesia immediately prior to randomisation Known contraindication to ketamine or morphine as per the SmPC Patient declines participation Known prisoner	
Study type	Interventional, blinded, randomised, individual assignment. Phase III trial
Date of first enrolment	10-Nov-21
Target sample size	446
Current recruitment	362
Recruitment status	Recruiting
Primary outcome	Outcome name: Sum of pain intensity difference score Metric/method of measurement: using a 0–10 numerical rating scale Timepoint: From randomisation to arrival at hospital
Key secondary outcomes	Effectiveness of pain relief and overall patient experience from randomisation to arrival at hospital Incidence of side effects and adverse events Resource use Longer term outcomes
Ethics Review	Status: Approved Date of Approval: 01/09/2020 Committee: West of Scotland Research Ethics Committee Contact: wosrec1@ggc.scot.nhs.uk

### Trial design and setting

This is a multi-centre, randomised controlled, double blinded trial comparing the clinical and cost-effectiveness of ketamine and morphine for severe pain following acute traumatic injury. It is a pragmatic, phase III trial working with two large NHS ambulance trusts with an internal pilot, aimed at mirroring existing practice of dealing with severe pain following acute traumatic injury. Participants will be followed up for 6 months. Adult patients (> = 16 years old) will be eligible for recruitment if they report severe pain following acute injury, in the pre-hospital environment and are determined by a paramedic to require parenteral morphine or equivalent.

The trial has been designed to determine if ketamine is superior to morphine as a prehospital analgesic. The prehospital phase ends once the patient arrives at hospital.

Randomisation will occur when the trial drug-pack is opened. The trial drug pack includes three ampoules of trial drug containing either ketamine of morphine. The ampoules of ketamine and morphine appear identical. The treating paramedic will not know which drug they are administering, hence the double blinding.

### Eligibility criteria

**Inclusion criteria**

1. Age ≥ 16
2. Patient reports a pain score ≥ 7/10 on a 0–10 NRS following acute traumatic injury
3. Intravenous or intraosseous access obtained
4. Determined by a paramedic to require IV morphine or equivalent

**Exclusion criteria**

1. Known or suspected pregnancy
2. Unable to articulate severity of pain using the 0–10 NRS
3. Lack of capacity due to a reason other than pain
4. Intravenous (IV) or intraosseous (IO) ketamine or opioid analgesia immediately prior to randomisation*
5. Known contraindication to either ketamine or morphine as per the published Summary of Product Characteristics (SmPC)
6. Patient declines participation
7. Known prisoner

*The trial is a pragmatic one and mirrors real life practice, hence why some analgesics e.g. paracetamol are not a justification for exclusion. Administration of ketamine or morphine is likely to significantly bias results hence why these two analgesics are excluded. Immediately before, here, refers to administration by a by a clinician responder who has arrived on scene prior to the arrival of the trial trained paramedic.

### Patient recruitment and consent

The study aims to recruit 446 participants. Potential participants will be identified by the attending paramedic and if eligible will be enrolled into the trial.

Acute severe pain disrupts cognitive function and may impair mental capacity. Furthermore, patients with severe pain require urgent treatment to relieve pain for humanitarian reasons as well as to reduce the physiological stress caused by severe pain. It may therefore be impractical to obtain written informed consent prior to treatment from either the patient or a personal legal representative as to do so would delay treating the patient’s pain.

Before recruiting any patient to the trial, the paramedic will provide the patient with brief verbal information about the trial by reading predefined text from an aide memoire and advising the patient of their intention to enrol the patient into the trial. At this time, the paramedic will not be seeking informed written consent, but will provide the patient the opportunity to decline participation in the trial. Written informed consent will be obtained from the patient, or their legal representative, by research paramedics at the earliest opportunity, once the initial emergency has passed.

Ethics approval was granted on 01/09/2020 by West of Scotland REC 1.

### Allocation sequence and randomisation

The randomisation sequence will be provided by the Programming Team at the Warwick CTU. Randomisation will be achieved by way of specially prepared, sequentially numbered treatment packs containing identical ampoules of either ketamine (intervention) or morphine (comparator). The content of the drug packs will be determined from a randomisation list prepared by the study programmer. The blinded block randomisation system will look to ensure a ratio of 1:1 control: intervention. The balance between arms at each site is handled by the ordering system that ensures a pre specified number of paired packs are delivered to each site. The block size is determined by the number of drugs in any given site batch order. Distribution of trial drug packs by the trial drug manufacturer will ensure equal proportions of ketamine (intervention) and morphine (comparator) are distributed to each participating site. Allocation will be concealed from study personnel, ambulance staff and patients.

Numbered study drug packs in a pre-randomised sequence, will be carried by participating ambulance paramedics. Randomisation will be achieved by opening the pack. This avoids the need for any randomisation procedures before recruitment which could delay patient treatment.

### Blinding

The packaging and the labelling of the IMP packs will conceal which trial drug is being used therefore the patient, attending clinicians, research paramedics and trial administration team will be blinded. Only the statistician and the programming team will be able to link the trial drug pack number to the allocation of ketamine or morphine.

### Intervention

**Ketamine** will be supplied in 2 ml glass ampoules containing 15 mg in 1 ml and supplied in numbered treatment packs containing 3 ampoules (up to 2 for administration and 1 in case of breakage).

**Morphine** will be supplied in 2 ml glass ampoules containing 10 mg in 1 ml and supplied in numbered treatment packs containing 3 ampoules (up to 2 for administration and 1 in case of breakage).

Trial IMP was manufactured and supplied by ModePharma Ltd, an MHRA licenced company who specialise in producing medications for clinical trials. The IMP is supplied in a standard white cardboard ampoule box.

### Dosing regime

The trial drug is prepared by diluting one ampoule of trial drug (either ketamine or morphine) with 9 ml of 0.9% sodium chloride in a 10 ml syringe. The trial drug is then administered by slow IV or IO injection, titrated to effect over five minutes (i.e. approximately 2 ml aliquots per minute), aiming to give the minimal effective dose.

If the patient still reports pain 5 min after receiving the first full syringe, the paramedic will prepare a second syringe of trial drug in the same manner. Further trial analgesia will be administered in 2 ml aliquots every 5 min until adequate pain relief is achieved, or all 10 ml of the second syringe has been administered.

The maximum volume of trial drug that can be administered is 20 ml (2 syringes), while the maximum dose that can be administered is 30 mg (two ampoules) of ketamine or 20 mg (two ampoules) of morphine. In standard practice, paramedics carry 2 × 10 mg in 1 ml ampoules of morphine. The maximum dose of morphine that a paramedic can administer is 20 mg (or two syringes). The rate of administration in the trial mirrors standard practice. Consequently, if a trial paramedic happened to open a drug pack containing morphine, then the patient would receive morphine in the exact same manner as standard practice.

The study is intended to compare morphine and sub-dissociative ketamine. Unfortunately, no morphine equivalent dose for ketamine has been published. Bredmose et al. [22] published a paper in a UK trauma population reporting a ketamine analgesic dose of 0.1 mg/kg. Currently, only specialist prehospital teams are able to administer ketamine for analgesia in the UK. The locally recommended dose is 0.2 mg/kg, which can be repeated up to a maximum of 0.5 mg/kg. Two studies by Motov [35, 36] used a dose of 0.3 mg/kg, but reported dissociative effects in some patients.

We wanted to minimise the likelihood of dissociative effects. Based upon mean UK adult weights (85.1 kg for males and 71.8 kg for females) we estimated that an average male would receive approximately 0.18 mg/kg from one syringe containing 15 mg ketamine, with the potential for further analgesia up to 0.36 mg/kg by administering the second syringe. Our approach of titration to effect (standard practice) should minimise the risk of larger dissociative doses being administered. More recent reviews by Riccardi et al. [37] and Sandberg et al. [38] have respectively recommended analgesic doses of 0.15–0.3 mg/kg and 0.1–0.2 mg/kg for ketamine. We therefore believe this dosing regime is consistent with the available evidence.

## Outcomes

Table 2 presents the timings of when study outcome measures will be collected. Table 3 details the definition and methods to calculate the study outcomes.

**Table 2:** Schedule of delivery of intervention and data collection

Visit	1	2	3	4
Visit Window (No. Weeks ± No. Days)	Baseline/Pre hospital/Hospital arrival	After hospital arrival	3 m (± 2w)	6 m (± 1 m)
Trial Information	✓	✓		
Informed consent		✓		
Randomisation	✓			
Vital signs	✓			
Inclusion/exclusion criteria	✓			
Intervention	✓			
Rescue analgesia	✓			
Quality of Life—EQ-5D-5L			✓	✓
Side effects & Adverse events	✓	✓	✓	✓
Questionnaire—BPI-SF			✓	✓
Questionnaire—CSRI			✓	✓
SPID	✓			
TOTPAR	✓			
Time to perceptible analgesia	✓			
Time to meaningful analgesia	✓			
Time to peak analgesia	✓			
Duration of analgesia	✓			
Patient Global Impression of Change	✓			
Resource use	✓	✓	✓	✓

**Table 3:** Outcome measure definitions

	Definition	Mitigations
*Primary outcome*		
Effectiveness of pain relief from randomisation to arrival at hospital as measured by Sum of Pain Intensity Difference (SPID) score (using a 0–10 numerical rating scale)	The SPID is measured using a weighted sum of the scores, as shown below: $SPID_{n} = \mathop \sum \limits_{i - 1}^{n} \left( {T_{i} - T_{i - 1} } \right)*PID_{i}$ *T*_*i*_ is the time in hours when observation *i* is taken *PID*_*i*_ is the difference in Pain Intensity (PI) scores from initial pain score to the pain score at time *T*_*i*_. The SPID looks to calculate the area under the curve of pain intensity different over time	
*Secondary outcomes*		
The Total Pain Relief (TOTPAR)	The TOTPAR is measured using a weighted sum of the scores, as shown below: ${TOTPAR}_{n}= \sum_{i-1}^{n}\left({T}_{i}-{T}_{i-1}\right)*{PAR}_{i}$ *T*_*i*_ is the time in hours when observation *i* is taken *PAR*_*i*_ is the pain relief score measured as defined below on a scale of 1 to 3 We measure minimal pain relief as 1.8 (1.7–1.9) change in pain score, much pain relief as 4.0 (3.9–4.1) and very much pain relief as 5.2 (5–5.4) Percentage changes defined as 20.3 (19–21.6), 44.4 (43.2–45.6), 56.1 (53.9–58.4) respectively	
Time to Perceptible Analgesia	Perceptible pain relief is defined as a 20% change in NRS pain score from the initial pain score. The time will be taken as time of perceptible analgesia minus time of first administration	If 20% change not achieved, we will score this as perceptible analgesia not achieved
Time to Meaningful Analgesia	Defined as a 44% change in NRS pain score from initial pain score. The time will be taken as time of meaningful analgesia minus time of first administration	If 44% change not achieved, we will score this as meaningful analgesia not achieved
Time to Peak Analgesia	Measured as the time when lowest NRS pain score, relative to initial pain score, is achieved minus time of first administration	
Duration of Analgesia	Measured as the time period in which patient pain scores have consecutively decreased or remained stationary (There may be different instances of this per patient)	
Requirement for Rescue Analgesia	We will record whether a patient has needed rescue analgesia and which analgesia was administered, Entonox, paracetamol, ibuprofen, or other	
Proportion of patients with pain intensity score below 4/10 on NRS scale	At hospital arrival the research paramedic will record a NRS pain score. We will provide the proportion, as a percentage, of each patient that achieved a score < 4/10	If there is no hospital arrival score, we will use the scores recorded in the ambulance journey
Vital Signs	At each observation time, the respiratory rate (bpm), oxygen saturations (%), heart rate (bpm), blood pressure (mmHg) and their Glasgow Coma Scale (GCS)	
Glasgow Coma Scale	Three subscales measuring eyes, verbal, and motor response of each patient. The scales are as such: Eyes 1–4 Verbal 1–5 Motor 1–6	
Global Impression of Change	Using a 7-point Likert scale. The options offered ranging from ‘very much improved’ to ‘very much worse’	
Side effects and adverse events	Measured in the following categories, ‘Airway’, ‘Respiratory’, ‘Cardiovascular’, ‘Neurologic’, and ‘other’	
Ambulance job cycle time	Time taken from arrival on scene to hospital arrival	
Number of ambulance resources	Number of doctors, paramedics, doctors, and vehicles attending scene	
Cumulative IMP doses administered	Total dose of IMP administered	
CT scan use	If patient had a CT scan and how many	
Hospital or ICU admission	Yes; no option if patient is admitted to hospital or ICU	
Length of stay in ED, ICU, or hospital	Classed as date and time of admission to date and time of discharge	
BPI-SF at 3 and 6 Months		
9 part self-reported form which allows us to monitor the severity of the patient’s pain and its effect on their daily life. Split into two sections, pain intensity and pain inference	9 part self-reported form which allows us to monitor the severity of the patient’s pain and its effect on their daily life	
Pain Intensity	The pain severity part assesses the pain of the patient at its worst, least, average and now	We can then determine the average of the 4 categories to determine pain intensity, however it is recommended that we present all 4 of the options
Pain Interference	The interference section measures the effect of pain in 7 different tasks, walking, work, mood, enjoyment of life, relations with others, and sleep	Measure as a mean if at least 4 of the sections have been completed

### Primary outcome

Effectiveness of pain relief from randomisation to arrival at hospital as measured by Sum of Pain Intensity Difference (SPID) score (using a 0–10 numerical rating scale(NRS). As this is a pragmatic trial, no fixed time interval was specified to record pain scores, however we did request that pain scores were documented regularly from the point of initial IMP administration to arrival at the hospital.

### Secondary outcomes

*Effectiveness of pain relief and overall patient experience from randomisation to arrival at hospital*

- Total Pain Relief (TOTPAR) score
- Time to perceptible analgesia
- Time to meaningful analgesia
- Time to peak analgesia
- Duration of analgesia
- Requirement for rescue analgesia
- Proportion of patients with a pain intensity score below 4/10 (0–10 numerical rating scale (NRS)) on arrival at hospital
- Vital signs (oxygen saturation, blood pressure, heart rate, respiration rate, Glasgow Coma Scale)
- Patient Global Impression of Change on arrival at hospital

*Incidence of side effects and adverse events*

- Airway: vomiting, aspiration, advanced airway management
- Respiratory: desaturation, need for ventilatory support
- Cardiovascular: arrhythmia, hypotension and hypertension
- Neurologic: sedation, excitatory movements, adverse behavioural reactions
- Other: nausea, allergic reaction

*Resource use*

- Ambulance job cycle time (scene arrival to arrival at hospital)
- Number of ambulance resources (technicians, paramedics, doctors and vehicles) in attendance
- Cumulative IMP doses administered
- CT scan use
- Hospital or ICU admission
- Length of stay ED, ICU, Hospital

*Longer term outcomes*

- Chronic pain using Brief pain inventory-short form (BPI-SF) at 3 & 6 months from randomisation
- Health-related quality of life measured using the EQ-5D-5L at 3 and 6 months from randomisation
- Cost-effectiveness expressed in terms of incremental cost per quality-adjusted life year (QALY) gained

### Sample size

In line with IMMPACT recommendations, our primary outcome reports Sum of Pain Intensity Difference (SPID). Reductions in pain severity can be reported as either change in NRS, the pain intensity difference (PID), or as a percentage change PID (%PID). The International Association for the Study of Pain has quantified clinically meaningful improvements in pain intensity. Depending on how severe the initial pain is, clinically important improvements in PID range from 1.3 to 5.2. Those with severe pain will need to experience a greater reduction in pain than those with mild pain to experience a clinically important reduction in pain. Similarly, improvements in %PID range from 20.1 to 56.1% depending on severity of pain. Previous studies have established that improvement in PID is equivalent to improvement in SPID [39].

The study has been powered to identify change in SPID calculated using the change in PID. To ensure our study is able to detect at least a 20% improvement in %SPID, regardless of baseline pain intensity, our sample size calculation is powered to detect 20% improvement in %PID, which in turn is equivalent to a 1 point difference (0–10 NRS) in effectiveness between morphine and ketamine.

Previous randomised controlled trials comparing ketamine and morphine have adopted a standard deviation of 3.0 [35, 36, 40, 41]. A review of existing prehospital studies identified that the average non-response/withdrawal rate was 14% [40, 42–45]. We therefore calculate our sample size assuming a standard deviation of 3.0, 1:1 randomisation, a power of 90%, significance level of 5% and a withdrawal/non-response rate of 15%.

Based on these estimates we calculate our trial will require a sample of 446 subjects, recruiting 223 to each arm of the study, to detect a 1 point difference on the NRS (range 0–10) in primary outcome between morphine and ketamine.

### Data analysis

The statistical analysis will follow the estimand framework [46]. Primary analysis will be by intention to treat. The primary outcome will be the SPID, a measure of the area under the curve of the pain score difference from baseline over time. The primary outcome will be analysed using a linear regression. Both unadjusted and adjusted (for important covariates) estimates and 95% confidence intervals for the treatment effect will be obtained. The adjusted estimates will form the basis for the primary analysis. Descriptive summaries of the outcomes will be presented as frequencies or means and medians. For the adjusted estimates, the covariates used will be age (< 60; ≥ 60 years), gender, weight and alternative parenteral IV paracetamol prior to randomisation (as a dichotomy split by yes or no). Age and gender have been chosen as covariates as these groups can experience pain differently, administration of IV paracetamol prior to randomisation is included as a covariate as it is an adjunctive treatment that may impact pain response. Weight is included as a covariate since different weight groups have different requirement for an adequate dose of IMP. For continuous secondary outcomes, analysis will be carried out in a similar way to the primary outcome. For categorical outcomes, logistic regression models will be used. Participant vital signs are recorded in a longitudinal format, for these outcomes we will use a mixed effect model.

Various intercurrent events have been identified for this trial, in line with the estimand framework [46], and approaches to dealing with these have been considered. For discontinuation of the allocated treatment and use of rescue analgesia, we will follow the treatment policy, where the data is analysed as observed. Compliance with administration of the trial drug as per the trial protocol will be monitored. If there is a noticeable degree of non-compliance, we will carry out a complier average casual effects (CACE) analysis [47, 48]. If sufficient data permits, sensitivity analysis will be conducted on a modified intention to treat population, excluding participants that were randomised but the drug was not given. Again, if sufficient data permits, participant deaths will be analysed using Pocock’s win ratio method, this allows death to be interpreted as a participant outcome and infer if the intervention is significantly better than the standard care having considered the clinical priority.

Item missingness is expected for the primary outcome due to the method of recording data used in the trial, however the primary outcome is a measure of the area under a curve, therefore if two pain measurements are recorded the primary outcome can still be measured. Imputation methods have been considered for missingness, but methods such as last observation carried forward are not reliable due to the variability of pain scores, and we cannot assume that pain scores are missing at random therefore multiple imputation is also not feasible. Analyses and template tables will be reported in a detailed statistical analysis plan for review and approval by the Data Monitoring Committee (DMC), prior to final statistical analysis of the data.

#### Subgroup analysis

We have selected the following subgroups to explore interactions relating to age, gender and the administration of intravenous paracetamol prior to randomisation. The primary outcome will be used as the dependent variable, interaction between the subgroup variable and treatment will be included as an independent variable. Linear regression models will be used to assess the subgroup effect, using interaction terms, subgroup by treatment, to measure the effect of each subgroup.

#### Data security

Participant data are being stored on a secure database in accordance with the Data Protection Act (1998). A unique trial identification number is used on all follow-up questionnaires. Warwick clinical trials unit does not receive nor process any personal identifiable data for this trial.

### Data collection and management

Source documents are where data are first recorded, and from which participants’ case report form (CRF) data are obtained. These include, but are not limited to, ambulance service records and hospital records (from which secondary outcome data will be collected from)*.* Patient eligibility and ambulance data will be collected from electronic patient records, whereas patient hospital data will be obtained from retrospectively from medical records. Follow up data is obtained from questionnaire packs posted out to participants that have consented to receive them.

On all trial-specific documents, other than the signed consent form, the participant will be referred to by the trial participant number/code, not by name. Data will be entered on to the trial database by the research team.

### Health economic evaluation

A health economic evaluation has been embedded into the PACKMaN trial. The economic evaluation will take the form of a within-trial cost-effectiveness analysis, conducted from the perspective of the UK NHS and personal social services [49]. Estimates of economic costs will capture resource use associated with the pre-hospital emergency response and broader utilisation of hospital and community-based health and social care services. Resource use in the pre-hospital stage will be extracted from trial case report forms completed by research paramedics. This will include the number of paramedic staff, technicians, doctors, and ambulance staff attending the patient, in addition to transport vehicle, duration of emergency response and cumulative morphine or ketamine doses administered, and medication for treatment of adverse events. Additionally, index admission hospitalisation data resource use will be extracted, this includes length of stay, and number of days receiving critical care and associated critical care level. Resource use questions completed by participants at each assessment point during the study follow-up will provide a profile of all other hospital inpatient and outpatient services, community health and social care encounters, prescribed medications, NHS supplies, time off work and out of pocket medical expenses. Health-related quality of life will be measured using the EQ-5D-5L at three and six months after randomisation. For ethical, logistical, and pragmatic reasons, it is not possible to capture baseline EQ-5D-5L measurements in patients suffering acute pain following trauma within this trial. This is not uncommon within trials in emergency and critical care settings [50]. The baseline analysis for the health economic evaluation will use a fixed baseline approach for EQ-5D-5L health utilities for all participants. This fixed value will be derived by mapping the ‘typical’ acute pain trauma case to the EQ-5D-5L using expert opinion. The sensitivity of this assumption will be tested within sensitivity analyses. Sensitivity analyses will include assigning different values to patients according to severity as determined by registration to the Trauma Audit and Research Network (TARN). TARN can be used as a proxy for severity as the most serious trauma patients will be registered onto TARN whilst less severe cases will not (non-TARN). We will then use expert opinion to estimate a baseline EQ-5D profile for both TARN and non-TARN patients. We will estimate QALY profiles for each participant over a six-month time horizon using the baseline-adjusted area-under-the curve method. We will fit a bivariate regression of costs and QALYs, with multiple imputation of missing data. We will estimate the incremental cost per QALY gained for the comparator interventions from incremental costs and incremental QALYs generated from the regressions. Cost-effectiveness estimates will also be generated for subgroups as specified in the health economics analysis plan.

The primary trial-based analysis will focus on the costs and QALYs accrued during the trial period. There is however potential for costs and benefits to accrue beyond the trial period. If outcomes have not converged by the 6 month timepoint we will consider extrapolating the results over a longer time horizon using a decision analytic model. This would involve combining the trial data with external sources to estimate the long-term cost-effectiveness of the intervention. Any costs and benefits accruing after the first year would be discounted at a rate of 3.5% per year and full probabilistic sensitivity analysis would be conducted in line with the NICE reference case [49]. A decision around the construction of a separate decision analytic model will be made following discussion between the health economists and the trial team following preliminary analysis of the data. This will be informed by considerations such as the conclusiveness and direction of within trial results. For example, if the control dominates the intervention and extrapolation would only increase the strength of this result then there is little need to extrapolate further as the intervention should be rejected. The decision to extrapolate cost-effectiveness will also take into account the availability and quality of external data to inform model parameter inputs.

### Adverse events

The trial is enrolling patients with acute traumatic injuries which may be immediately life threatening, or result in hospitalisation, persistent or significant disability/incapacity and or death. Potential adverse events are captured on the case report form and investigated by the research paramedic in the first instance. The research paramedic passes the results of their investigation to the PI to determine if the event is an adverse event or not. The trial team are then informed of the outcome as required. In addition, hospital clinicians are able to report clinical concerns to the research paramedic for review that will similarly be reported via the PI to the CI and onward as necessary.

Clinically predictable side effects will be captured but may not be classified as adverse events. For example, morphine is known to cause respiratory depression. A reduction in respiratory rate will be captured on case report forms. If no intervention is required, this will not be classified as an adverse event. However, if the treating paramedic has to assist ventilations of administer naloxone, then this would be classified as an adverse event.

The following adverse events are captured on the case report form as secondary outcomes. If deemed serious they will also be recorded and reported using the SAE form.

- Airway: vomiting, aspiration, advanced airway management
- Respiratory: desaturation, need for ventilatory support
- Cardiovascular: arrhythmia, hypotension and hypertension
- Neurologic: sedation, excitatory movements, adverse behavioural reactions
- Other: nausea, allergic reaction

Serious adverse events which are not related to the acute traumatic injury, or are complications resulting from the IMP administration to 30 days post trial drug will be reported to the PACKMaN Trial team as soon as possible and within 24 h of the research staff becoming aware of the event.

#### Reporting

Results from the PACKMaN trial will be reported to a trial registry within 12 months of a database lock.

**Fig. 1:**
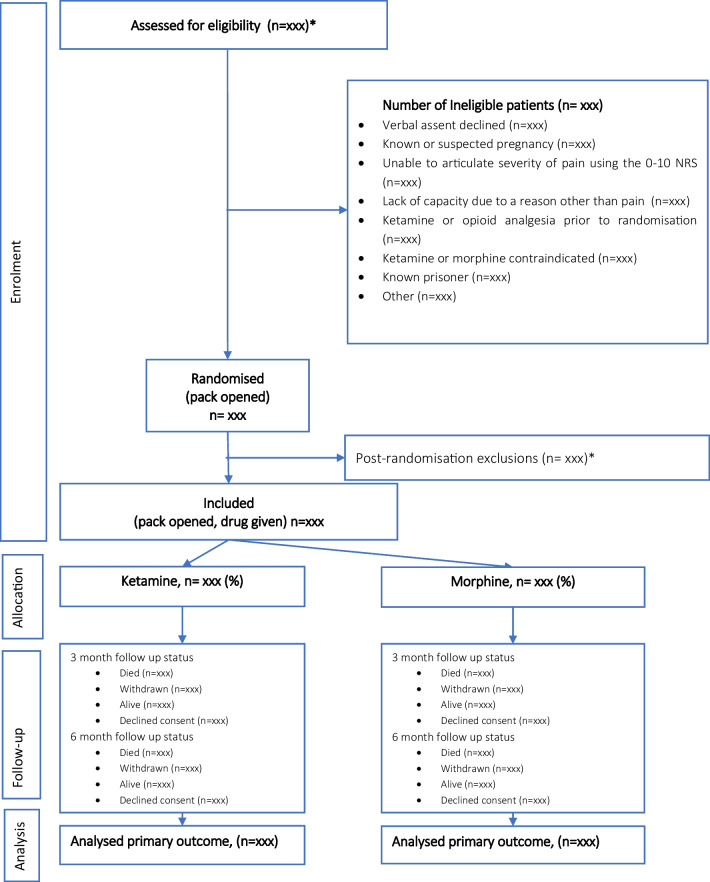
CONSORT diagram

The trial will be reported in accordance with the Consolidated Standards of Reporting Trials (CONSORT) guidelines (Fig. 1) [51].

#### Dissemination

Our dissemination strategy will target policy makers, commissioners, trauma networks, ambulance services, healthcare providers, academic audiences, patients and the public, charities and advocacy groups. It will include presentations at national and international conferences. We will submit publications to open access peer reviewed journals, develop a lay summary and infographic of the research findings. We will work with our patient and public partners to develop patient stories which effectively communicate key messages from the study. We will publicise via press releases to established media contacts and use our website, blog, Facebook page and Twitter feed to communicate our findings. Our research will support the development of an evidence-based pain management guideline for paramedics by NHS ambulance services. It will improve healthcare quality for patients with severe pain following trauma by engaging clinicians, patients, ambulance services and policy makers to provide better care, by reducing variation in practice and optimising the use of limited health resources.

#### Data monitoring committee

Professor Siobhan Creanor (Chair), Professor Julia Williams, Dr Charlotte Small.

#### Trial steering committee

Dr Fionna Moore (Chair), Tim Edwards, Andy Collen, Caroline Leech, Jonathan Bishop, Claire Hulme, Maria Devlin.

#### Collaborators

PACKMaN Study Group: chief investigator: Professor Gavin Perkins. Co-chief investigator: Dr Michael Smyth. Co-investigators (Grant holders): Dr Joyce Yeung, Professor Ranjit Lall, Dr Gordon Fuller, Professor Stavros Petrou, Dr Allison Walker, Dr Julian Mark, Duncan Buckley. Senior project manager: Kath Starr. Trial co-ordination/administration: Dr Hannah Noordali. Research fellows/assistants: Felix Michelet (medical statistics), Kamran Khan (Health economics). Patient representative: Duncan Buckley. Trial statistician: Professor Ranjit Lall. Health economist: Professor Stavros Petrou. Intervention development: MODEPHARMA Limited. Data programming team: Ade Willis, Chockalingam Muthiah.

#### Data sharing

The trial statisticians and DMEC will have access to the dataset for the analysis of trial outcomes. Once the main analyses have been undertaken, deidentified individual participant data will be available to principal and other investigators subject to approval of data analysis plans by the TSC and compliance with the University of Warwick SOPs on Data Management and Sharing. We will comply with Data Sharing Policies that may be instituted by the NIHR during the lifetime of the project.

#### Ethics approval

West of Scotland REC 1 approved 01/09/2020.

#### Provenance and peer review

The study was independently peer reviewed as part of the funding application to the NIHR.

## Data Availability

Not applicable.
